
JOURNAL INFORMATION
==============================
NLM Title Abbreviation: Inter Econ
Journal ID: Inter Econ
NLM Journal ID: 101086399

Inter Economics

ISSN: 0020-5346

ARTICLE INFORMATION
==============================
PMCID: PMC8341241
PMID: 34376871
DOI: 10.1007/s10272-021-0990-9
Article ID: 990
Article version: 1
Subjects: Letter from America

Bidenomics: Content and Prospects

Eichengreen Barry eichengr@econ.berkeley.edu

grid.47840.3f 0000 0001 2181 7878 Department of Economics, University of California, Berkeley, 30 Evans Hall, MC #3880, Berkeley, CA 94720-3880 USA
Electronic publication date: 2021 Aug 5
Print publication date: 2021
Volume: 56
Issue: 4
First page: 243
Last page: 244
Copyright: © The Author(s) 2021
License: This article is distributed under the terms of the Creative Commons Attribution 4.0 International License (https://creativecommons.org/licenses/by/4.0/).
Open Access funding provided by ZBW — Leibniz Information Centre for Economics.
License URL: https://creativecommons.org/licenses/by/4.0/

issue-copyright-statement: © The Author(s) 2021

==============================

Six months into Joe Biden's presidency, a Google search for "Bidenomics" returns 256,000 hits. Unfortunately, such a search does not tell us the meaning of the term, or even whether a coherent concept exists. At root, President Biden and his team envisage a more expansive economic role for government. Biden was elected to the Senate in 1972, having come of political age in the era of Lyndon Johnson's "Great Society" of spending programs on education, healthcare, urban renewal and anti-poverty. Biden's infrastructure proposal envisaged $2.3 trillion of new spending on roads, bridges, broadband and climate change abatement. This was twinned with his $1.8 trillion American Families Plan for healthcare, childcare, eldercare and education programs.

Over the summer, the infrastructure package was downsized to $600 billion of new spending. The revision was deemed necessary to bring Republican legislators on board and retain the support of moderate Democrats from heavily Republican states, such as Senator Joe Manchin of West Virginia.

Infrastructure renewal is something on which both parties in principle can agree. It will bring jobs to underserved communities. It appeals to passengers held captive on planes at airports operating above capacity. But embarrassment over the condition of America's airports is of long standing, as is awareness that some communities and regions have inadequate access to broadband and other infrastructure. What is new is recognition that infrastructure inadequacies handicap the U.S. in competition with China, now seen by Democrats and Republicans alike as an economic rival and geostrategic threat. This concern is what makes possible support for an infrastructure bill by members of a Republican Party temperamentally opposed to spending on anything other than military and police.

Paying for this infrastructure initiative is another matter. Republicans oppose higher taxes on corporations, capital gains and the wealthy. The bipartisan deal therefore foresees financing infrastructure spending through public-private partnerships, where future revenues from, *inter alia*, toll roads are sold off to investors in return for underwriting construction costs.

This, clearly, is business as usual rather than a revolution in the role of the state, though it represents a healthy turn away from the doctrinaire belief that the market solves all problems. But it is revealing that most climate change measures had to be stripped out of the infrastructure deal in order to garner Republican support (and retain the support of Senator Manchin). This does not exactly indicate that the United States has entered a new era of enlightened bipartisanship and reality-based policy.

More revolutionary would be the creation and expansion of federal programs providing universal preschool, subsidized childcare, nutritional assistance for children, free community college and home-based care for the elderly, as envisaged by Biden's American Families Plan and also by a more ambitious bill tabled by the progressive Senator Bernie Saunders. These initiatives have been likened to Franklin Roosevelt's New Deal, which created unemployment insurance, a minimum wage and federally funded pensions. They would be a sea change for a country traditionally reluctant to contemplate anything resembling the European welfare state.

Whether any of this will happen remains unclear. Some view the question through the lens of successive American economic and political orders. First there was the "New Deal Order", when it was taken for granted that the government would provide public goods and services. In the 1980s, this gave way to the "Neoliberal Order," when Ronald Reagan ushered in an era of limited government and market fundamentalism. Perhaps the pendulum is now swinging back, as Biden imagines, toward a "New New Deal Order."

Certainly the pandemic has alerted many Americans to the precariousness of their lives. It shone a light on the role of government in protecting people against risks from which they cannot protect themselves. In this sense, COVID-19 may have the same effect as the Great Depression, the economic catastrophe that bred support for the original New Deal.

That said, the 1930s Depression worsened for four full years, whereas recovery from the COVID-19 crisis commenced in less than one. To be sure, the pandemic has been a public health catastrophe for the United States. No doubt, its memory will engender support for better funding for the Centers for Disease Control and other public health agencies. Less certain is that it will weaken the ethos of "rugged individualism" that is baked into America's political and social DNA, or that it will beget meaningful support for a more expansive welfare state. An obvious litmus test was the referendum in California last November on whether gig workers should be classified as employees rather than independent contractors, requiring rideshare firms to pay payroll taxes and entitling workers to unemployment relief and related employee benefits. The proposition was roundly defeated at the polls despite being decided at the height of the pandemic.

FDR, when seeking to push through the New Deal, faced resistance from Republicans who saw his programs then, like Biden's now, as creeping socialism, and from Southern Democrats opposed to federal interference in local affairs. He overcame opposition from the latter by giving state and local officials control of federal funds, which Southern politicians used to favor their white constituents. FDR agreed to exclude farm workers from the Fair Labor Standards Act in order to gain the vote of Southern Democrats representing farmers who relied on cheap Black labor. He allowed Southern Democrats to dictate that the cheap electricity produced by the Tennessee Valley Authority would flow to racially segregated communities. Such was the regrettable price of Roosevelt's economic revolution.

Leading to the question of what quid pro quo Biden will have to offer to win over skeptics of his American Families Plan. Support of a bare majority of 51 Senate Democrats (including the vice president's vote) can presumably be maintained for a de minimis version of the bill, which could be passed using the process known as "reconciliation". But getting conservatives to support a more ambitious version will require more.

The debate over election reform offers a hint. The issue here is Republican-dominated state legislatures limiting access to the ballot for disadvantaged, primarily Black communities that vote Democrat. The Biden Administration chose to abandon more far-reaching federal legislation overriding such measures in favor of Senator Manchin's more limited bill as the price of securing Manchin's support for the infrastructure deal. We may see more such horse trading when the Senate turns to Biden's Families Plan.

But there are two differences between the 1930s and today. First, Biden is more committed than FDR to the cause of racial justice, or so it seems. Second, whereas 1930s Northern liberal opposition to racial discrimination was only skin deep, allowing Northern Democrats to cast a blind eye on Southern segregationism, today's progressive Democrats are not prepared to ignore electoral injustice along racial lines. Social compromises designed to attract Republican and Conservative Democratic votes for Biden's economic program will only peel off progressive support.

Bidenomics remains a work in progress; the preceding is all subject to change. But if this diagnosis is accurate, it suggests that Bidenomics faces a narrow path, and that it may end up being less than a New New Deal.

Barry Eichengreen, University of California, Berkeley, USA.

